# Supplementary material for: Content-rich biological network constructed by mining PubMed abstracts
Source: BMC Bioinformatics. 2004 Oct 8;5:147. doi: 10.1186/1471-2105-5-147 (PMC528731; doi:10.1186/1471-2105-5-147)
Supplement: Additional File 5 — The original Chilibot query results of the term "long-term potentiation (LTP)" and 22 other terms, limiting the latest references analyzed to the years 1990, 1995, 2000, and 2004. [file 1471-2105-5-147-S5.bz2 › chilibotAdditionalFile5/ltp1995/html/TAU_CAMKII.html]

 


 **TAU** and **CAMKII** 
  
Found 6 abstracts in PubMed,  **6 abstracts were retrieved and analyzed**.  


---

 Search Google  |
 PDF files only 
|  EDU domain only 

---

**Interactive relationship** (e.g. stimulation, inhibition, etc)

- Calcium calmodulin dependent protein kinase II  [ **CAMKII** ]  CaM kinase II has been shown to phosphorylate  **tau**  in vitro in such a way to decrease its electrophoretic mobility.  Ref: 1312209 Brain Res Mol Brain Res, 1992
- **tau**  self association was also significantly decreased by prior phosphorylation with calcium calmodulin dependent protein kinase II  [ **CAMKII** ] .  Ref: 7861153 J Neurochem, 1995
- Both cAMP PK and  **CaMKII**  readily phosphorylated  **tau**  and slowed the migration of  **tau**  on sodium dodecyl sulfate containing polyacrylamide gels.  Ref: 1331323 J Neurochem, 1992
- Two dimensional phosphopeptide maps and sequential phosphorylation experiments indicate that cAMP PK phosphorylates sites in the human  **tau**  isoforms that are phosphorylated by  **CaMKII** , as well as unique sites that are not phosphorylated by  **CaMKII** .  Ref: 8384512 Brain Res, 1993
- Both cAMP dependent protein kinase cAMP PK and calcium calmodulin dependent protein kinase II  **CaMKII**  readily phosphorylated the three human  **tau**  isoforms, although cAMP PK phosphorylated them to a significantly greater extent than  **CaMKII** .  Ref: 8384512 Brain Res, 1993
- Two dimensional phosphopeptide maps indicate that cAMP PK phosphorylates a site or sites on  **tau**  that are phosphorylated by  **CaMKII** , as well as a unique site or sites that are not phosphorylated by  **CaMKII** .  Ref: 1331323 J Neurochem, 1992
- However, cAMP PK phosphorylated  **tau**  to a significantly greater extent than  **CaMKII**  1.5 and 0.9 mol of 32P mol of  **tau** , respectively, and phosphorylation of  **tau**  by cAMP PK resulted in a greater shift to a more acidic, less heterogeneous pattern on two dimensional nonequilibrium pH gradient gels compared with  **CaMKII**  phosphorylation.  Ref: 1331323 J Neurochem, 1992

**Parallel relationship** (e.g. studied together, co-existance, homology, etc.)

- In this study we have evaluated six non PDPKs cyclic AMP dependent A kinase, calcium phospholipid dependent C kinase, casein kinase 1 CK 1, casein kinase 2 CK 2, calcium calmodulin dependent protein kinase II  [ **CAMKII** ] , and calcium calmodulin dependent protein kinase from rat cerebellum for their abilities to induce Alzheimer like epitopes on  **tau** .  Ref: 7532213 J Neurochem, 1995
- In situ hybridization of calcium calmodulin dependent protein kinase II  [ **CAMKII** ]  and  **tau**  mRNAs.  Ref: 1312209 Brain Res Mol Brain Res, 1992
- The effects of cyclic AMP dependent protein kinase cAMP PK or calcium calmodulin dependent protein kinase II  **CaMKII**  phosphorylation on the binding of bovine  **tau**  to tubulin and calpain mediated degradation of  **tau**  were studied.  Ref: 1331323 J Neurochem, 1992
- **CaMKII**  phosphorylation of  **tau**  did not alter either of these parameters.  Ref: 1331323 J Neurochem, 1992
